# Analysis of Pediatric Secondary Glaucoma Patients Treated with Micropulse Transscleral Cyclophotocoagulation (MP-TSCPC)

**DOI:** 10.3390/life16030384

**Published:** 2026-02-27

**Authors:** Bogumiła Wójcik-Niklewska, Karolina Pańczyk, Karina Dzięcioł, Nikola Oleksyk, Zofia Oliwa, Mariola Dorecka, Dorota Wyględowska-Promieńska, Adrian Smędowski

**Affiliations:** 1 Department of Pediatric Ophthalmology, Faculty of Medical Sciences in Katowice, Medical University of Silesia, 40-514 Katowice, Poland; asmedowski@sum.edu.pl; 2 Department of Pediatric Ophthalmology, Professor Kornel Gibiński University Clinical Center, Medical University of Silesia, 40-514 Katowice, Poland; 3 Students Scientific Society, Department of Ophthalmology, Faculty of Medical Sciences in Katowice, Medical University of Silesia, 40-514 Katowice, Poland; karolina.panczyk19@gmail.com (K.P.); karina.dzieciol@o2.pl (K.D.); nikolaoleksyk@gmail.com (N.O.); zocha2002@gmail.com (Z.O.); 4 Department of Ophthalmology, Faculty of Medical Sciences in Katowice, Medical University of Silesia, 40-514 Katowice, Poland; marioladorecka@wp.pl (M.D.); dwygledowska@sum.edu.pl (D.W.-P.); 5 Department of Ophthalmology, Professor Kornel Gibiński University Clinical Center, Medical University of Silesia, 40-514 Katowice, Poland; 6 The Laboratory for Translational Research in Ophthalmology, Department of Opthalmology, Faculty of Medical Sciences in Katowice, Medical University of Silesia, 40-514 Katowice, Poland; 7 GlaucoTech Co., Ltd., 40-514 Katowice, Poland

**Keywords:** pediatric glaucoma, secondary glaucoma, micropulse transscleral cyclophotocoagulation, intraocular pressure

## Abstract

Background: Secondary glaucoma in children results from congenital or acquired ocular abnormalities, systemic diseases, or syndromes. These conditions impair aqueous humor outflow despite an open iridocorneal angle, causing elevated intraocular pressure (IOP). Micropulse transscleral cyclophotocoagulation (MP-TSCPC) reduces aqueous humor production by targeting the ciliary body and restores the aqueous humor’s circulation balance. The aim of the study was to evaluate the safety and efficacy of MP-TSCPC in pediatric secondary glaucoma. Methods: This retrospective study included 59 children who underwent MP-TSCPC procedures. The mean age was 7.2 years (range 4 months–17 years). Data on IOP, prior glaucoma treatments, medication use, and adverse events were analyzed. The mean follow-up was 10.4 months. Results: The mean preoperative IOP was 34.0 mmHg, which significantly decreased to 25.8 mmHg after MP-TSCPC, representing a mean reduction of 20.8% (*p* < 0.0001). Satisfactory IOP lowering was achieved in 69.6% of procedures. Eyes without prior glaucoma surgery showed a numerically greater IOP reduction (22%) compared to previously treated eyes (19%), though the difference was not statistically significant (*p* = 0.628). Among repeated MP-TSCPC treatments, 57.1% were successful, with a mean IOP reduction of 7.3%. The mean number of glaucoma medications decreased significantly from 2.42 to 2.02 (*p* = 0.0002). A sustained reduction in medication use was observed in 33.3% of cases. Conclusions: MP-TSCPC effectively lowers IOP in pediatric secondary glaucoma and has a favorable safety profile. The option for repeated treatments and reduced medication needs supports its use as a less invasive alternative to conventional surgery.

## 1. Introduction

Secondary glaucoma in children is a heterogeneous group of diseases resulting from congenital or acquired ocular structural abnormalities, systemic diseases, and syndromes that can lead to irreversible vision loss [1]. It occurs as a complication of primary ocular diseases, such as chronic uveitis [2], traumas [3], tumors [4], or complications following ophthalmic surgeries [5]. The mechanism of its development involves impaired aqueous humor outflow, leading to increased intraocular pressure (IOP) and subsequent optic nerve damage [6]. In pediatric patients, secondary glaucoma may also be associated with developmental anomalies, such as aniridia or Axenfeld–Rieger or Peters syndrome [7], as well as with systemic diseases including Sturge-Weber syndrome [8,9]. Moreover, early-onset glaucoma has been associated with genetic mutations affecting anterior segment development [10]. These associations highlight the importance of early diagnosis and personalized treatment strategies. The primary treatment methods for secondary glaucoma in children include pharmacotherapy, laser therapy, and various surgical interventions [11,12]. The choice of treatment depends on the underlying cause and severity of the disease. Pharmacotherapy involves the use of IOP-lowering medications. Common medication groups include prostaglandin analogs [13,14], β-blockers [15,16], carbonic anhydrase inhibitors [17], α-2 agonists, parasympathomimetics, miotic agents, more recently Rho-kinase inhibitors [18] and nitric oxide-donating medications [19].

Laser therapy, such as cyclophotocoagulation, aims to reduce aqueous humor production by targeting the ciliary body epithelium. Laser trabeculoplasty, including argon laser trabeculoplasty, selective laser trabeculoplasty [20], and micropulse laser trabeculoplasty, may also be used in selected cases [21]. Surgical interventions, such as trabeculectomy, deep sclerectomy, canaloplasty, or drainage device implantation, are considered when other treatments prove insufficient [22].

Micropulse transscleral cyclophotocoagulation (MP-TSCPC) with a diode laser is a modern treatment method for secondary glaucoma [23]. It applies short laser pulses to the ciliary body through the sclera, reducing aqueous humor production and IOP. Unlike conventional continuous-wave transscleral cyclophotocoagulation (CW-TSCPC), in which laser energy is delivered in a continuous manner causing sustained thermal coagulation of the ciliary body, the micropulse mode delivers laser energy in repetitive short pulses separated by rest intervals. This delivery pattern is defined by a duty cycle, which represents the percentage of time during which the laser is actively emitting energy within each cycle. The “on” periods are followed by “off” periods that allow thermal relaxation of the treated tissue, thereby limiting excessive heat accumulation and minimizing collateral tissue destruction.

In clinical practice, micropulse treatment typically uses a predefined duty cycle (commonly around 31.3%), with power and exposure time adjusted according to patient age and ocular condition. The intermittent energy delivery results in a more controlled and subthreshold thermal effect compared to continuous-wave treatment, which may reduce the risk of complications such as hypotony, inflammation, or phthisis bulbi.

MP-TSCPC also increases aqueous humor outflow through the conventional pathway by inducing contraction of ciliary muscle fibers, leading to trabecular meshwork and Schlemm’s canal remodeling. Additionally, outflow through the suprachoroidal space is enhanced via matrix metalloproteinase activation, which expands the extracellular space and opens the suprachoroidal pathway [24,25]. The procedure is minimally invasive, short, and repeatable when necessary. Studies confirm its effectiveness in reducing IOP in secondary glaucoma [26,27].

The aim of this study is to analyze the safety and the effectiveness of MP-TSCPC in children with secondary glaucoma.

## 2. Materials and Methods

This single-center case–control study included 59 pediatric patients with secondary glaucoma, involving a total of 68 eyes treated with the MP-TSCPC procedure between January 2020 and December 2024. A total of 102 MP-TSCPC procedures were performed, as some eyes required repeated treatments. Patients’ age ranged from 4 months to 17 years (204 months), with a mean age of 86.39 months (median: 60, SD: 65.33). The mean follow-up was 10.4 months (median: 7.89, SD: 10.16), during which an average of five postoperative control visits were conducted.

The analyzed data included general demographics (age and gender) and clinical information, pre- and post-procedure findings, IOP values, details regarding previous anti-glaucoma surgeries (count and type), use of glaucoma medications (count and type), and post-procedure adverse events.

Genetic testing was not routinely performed in this cohort. However, clinical evaluation allowed assessment of phenotype–genotype correlations when features suggested a possible genetic origin.

Data were analyzed using the R statistical environment (RStudio, 2025.09.1+401), with data processing performed using the dplyr, ggplot2, and tidyr packages. Continuous variables were summarized as means with standard deviations or medians with interquartile ranges, as appropriate. Normality of distributions was assessed prior to analysis using the Shapiro–Wilk test. The Shapiro–Wilk test indicated that the majority of continuous variables, including baseline IOP values and follow-up measurements, did not follow a normal distribution (*p* < 0.05). Therefore, non-parametric methods were primarily applied for comparisons involving these variables. Parametric tests were used only when the assumption of normality was met. Paired comparisons were conducted using the paired Student’s *t*-test when normality assumptions were met, while between-group comparisons were performed using Welch’s two-sample *t*-test for parametric data and the Kruskal–Wallis test for non-parametric comparisons between multiple groups. Correlations between variables were evaluated using Spearman’s rank correlation coefficient when appropriate. A two-sided *p* value < 0.05 was considered statistically significant.

All procedures were performed using the Cyclo G6 laser system (IRIDEX) with the MicroPulse P3 probe (Iridex Corporation, Mountain View, CA, USA). The laser settings were as follows: power of 1200 mW, duty cycle of 31.3%, and duration of each pulse was 300 ms, with a treatment duration of 4 s per spot. All procedures were performed by the same experienced physician, ensuring consistency in the execution of the technique. The primary outcome was the reduction in IOP after MP-TSCPC, analyzed separately for each eye respecting any previous glaucoma interventions. Secondary outcomes included changes in glaucoma medication use and the effectiveness of repeated MP-TSCPC sessions.

The study received approval of the Bioethical Committee of the Medical University of Silesia (opinion number PCN/CBN/0052/KB/265/22).

## 3. Results

In the analyzed group of patients with secondary glaucoma treated with MP-TSCPC, the most common recorded condition was uveitis, observed in 50 eyes. The second most frequent factor was aphakia after congenital cataract surgery, diagnosed in 47 eyes.

Overall, the cohort included a broad spectrum of pediatric secondary glaucoma phenotypes, encompassing both acquired and congenital forms. Developmental anterior segment anomalies were represented by Axenfeld-Rieger anomaly, aniridia, and Peters anomaly. Other less common etiologies included post-traumatic glaucoma, congenital optic nerve or choroidal coloboma, and syndromic conditions such as Sturge–Weber syndrome. Although no patients had genetically confirmed diagnoses, clinical evaluation allowed assessment of phenotype–genotype correlations when features suggested a possible genetic origin.

A considerable number of cases were also associated with aphakia following complicated cataract surgery (17 eyes), pseudophakia after congenital cataract surgery (11 eyes), corneal dystrophy (11 eyes) and band keratopathy (7 eyes).

Less common factors were microphthalmia (6 eyes), microcornea (5 eyes), genetic juvenile glaucoma (5 eyes), status post intravitreal hemorrhage (5 eyes), aphakia after developmental cataract surgery (5 eyes), and persistent fetal vasculature (5 eyes).

**Figure 1:**
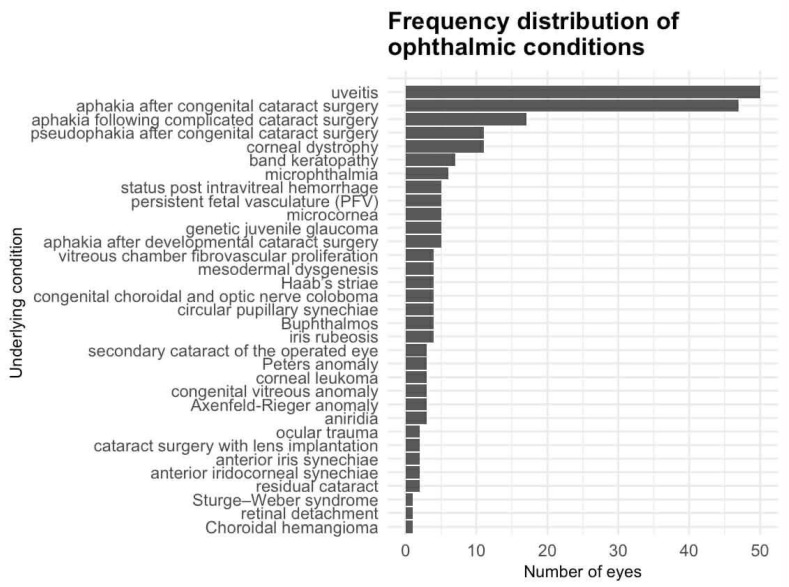
Frequency distribution of ophthalmic conditions in the studied cohort.

Factors occurring in 4 eyes each included congenital choroidal and optic nerve coloboma, Haab’s striae, mesodermal dysgenesis, buphthalmos, iris rubeosis, and vitreous chamber fibrovascular proliferation. Conditions documented in 3 eyes each were Axenfeld-Rieger anomaly, corneal leukoma, circular pupillary synechiae, secondary cataract of the operated eye, aniridia, Peters anomaly, and congenital vitreous anomaly. The least common causes (≤2 eyes each) were ocular trauma, residual cataract, cataract surgery with lens implantation, anterior iridocorneal synechiae, anterior iris synechiae, retinal detachment, Sturge–Weber syndrome, and choroidal hemangioma. Among 68 eyes, many of them presented coexisting multiple pathologies (Figure 1).

Forty-nine procedures (48.0%) were performed on eyes with a history of prior glaucoma surgery, while 53 procedures (52.0%) involved surgery-naive eyes. Among previously treated eyes, the most common prior surgery was trabeculectomy with iridectomy (24 procedures, 23.5%), followed by trabeculectomy with mitomycin C (6 procedures, 5.9%). Other interventions included diode laser transscleral cyclophotocoagulation (10 procedures, 9.8%) and cyclocryotherapy (4 procedures, 3.9%).

**Figure 2:**
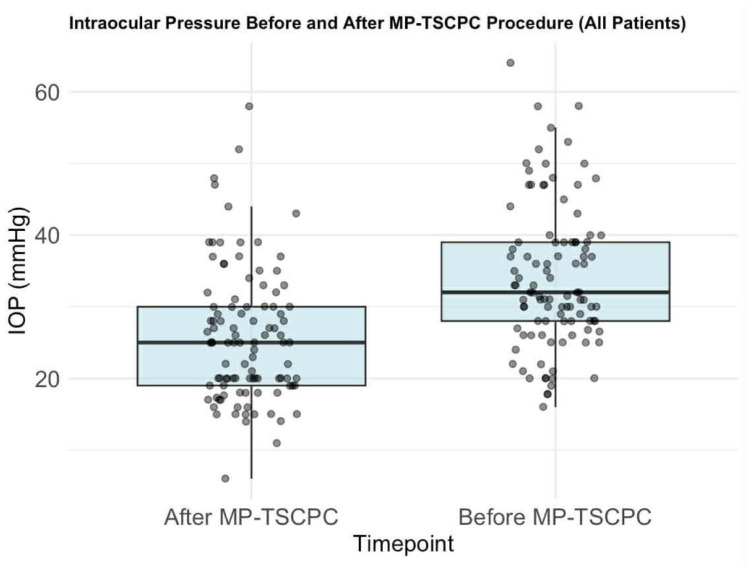
Intraocular pressure before and after MP-TSCPC procedure (all patients). Individual dots represent single-patient IOP measurements; darker dots indicate overlapping observations. Boxes indicate the interquartile range (IQR), the horizontal line within each box represents the median, and whiskers denote the data range.

**Figure 3:**
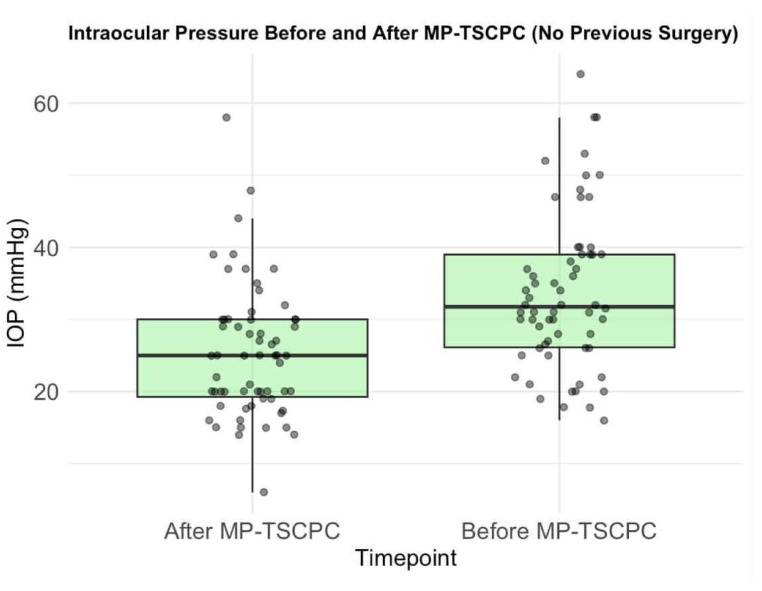
Intraocular pressure before and after MP-TSCPC (no previous surgery). Individual dots represent single-patient IOP measurements; darker dots indicate overlapping observations. Boxes indicate the interquartile range (IQR), the horizontal line within each box represents the median, and whiskers denote the data range.

**Figure 4:**
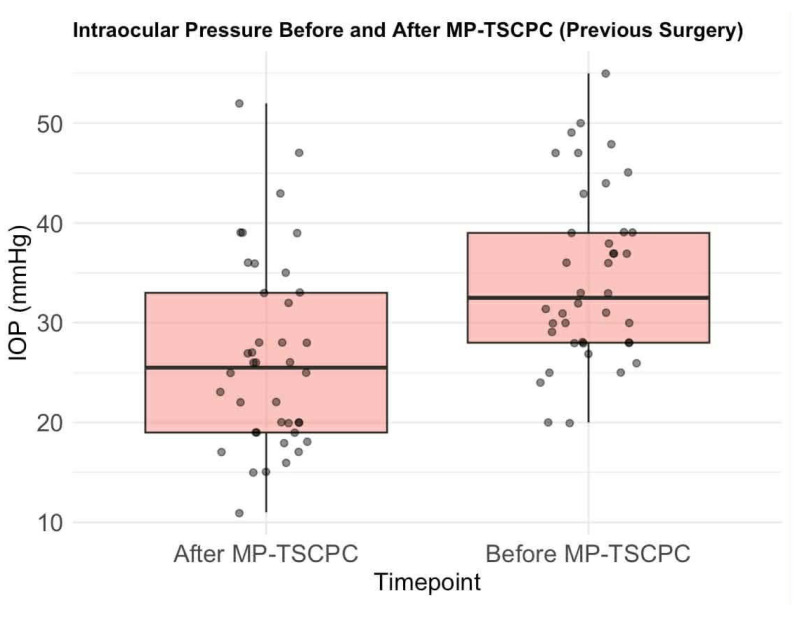
Intraocular pressure before and after MP-TSCPC (previous surgery). Individual dots represent single-patient IOP measurements; darker dots indicate overlapping observations. Boxes indicate the interquartile range (IQR), the horizontal line within each box represents the median, and whiskers denote the data range.

The mean preoperative IOP was 34.0 mmHg (median: 32, SD: 10). Following MP-TSCPC, mean IOP significantly decreased to 25.8 mmHg (median: 25, SD: 9.2), reflecting an average reduction of 20.8% (median: 26.5%, SD: 28.6%; *p* < 0.0001, paired *t*-test) (Figure 2). Eyes without prior glaucoma surgery showed a greater mean IOP reduction (22%, SD: 26.2%) (Figure 3) compared to previously treated eyes (19%, SD: 32.2%) (Figure 4), though the difference was not statistically significant (Welch Two Sample *t*-test *p* = 0.6282). Satisfactory IOP reduction occurred after 72 procedures (69.6%), while 30 procedures (30.4%) did not result in IOP value improvement. Among 28 repeated MP-TSCPC treatments, a satisfactory outcome was achieved in 16 cases (57.14%), with a mean IOP reduction of 7.3% (median: 14%, SD: 28.5%). These procedures were recommended in cases where previous glaucoma treatments (such as trabeculectomy or pharmacological therapy) had failed to adequately control IOP, or when the patient’s condition worsened despite earlier interventions. The decision to perform repeated MP-TSCPC was made based on high preoperative IOP and the failure of prior surgeries to control pressure effectively. The mean preoperative IOP for these repeated procedures was 34.0 mmHg (SD: 10). Postoperatively, the mean IOP was approximately 31.5 mmHg, reflecting a 7.3% reduction. Although this reduction was relatively modest, it was considered clinically relevant for patients with refractory glaucoma, as it resulted in stabilization of IOP and a reduction in the need for further surgical interventions or additional medication.

Patients without prior surgical interventions demonstrated a stronger response to treatment. Among cases with available medication data (*n* = 96), the mean number of glaucoma medications decreased from 2.42 (median: 2, SD: 0.69) preoperatively to 2.02 (median: 2, SD: 0.99) postoperatively. The mean reduction in medication use was 0.40 drugs (SD: 1.07, 95% CI: 0.18–0.61). This decrease was statistically significant based on the paired Wilcoxon signed-rank test (*p* = 0.000249).

A sustained reduction in glaucoma medication use was observed after 32 procedures (33.3%), no change after 47 procedures (49.0%), and a sustained increase after 17 procedures (17.7%) in which mean IOP remained largely unchanged, measuring 34.0 mmHg preoperatively and 34.75 mmHg postoperatively.

**Figure 5:**
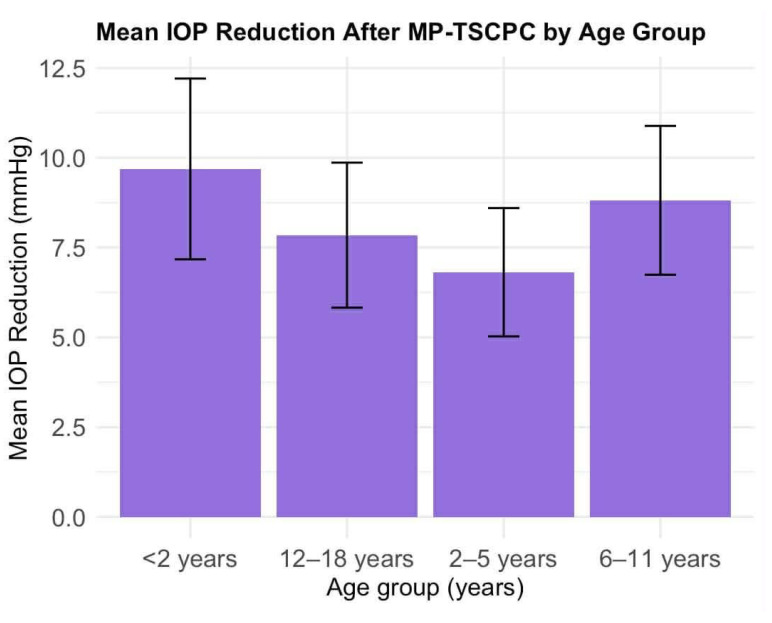
Mean IOP reduction after MP-TSCPC by age group.

Age group analysis showed the highest mean percentage IOP reduction in children aged 6–11 years (mean reduction 24.1%, SD 23.8%; *n* = 18), followed by adolescents aged 12–17 years (21.6%, SD 28.6%; *n* = 28). Younger children exhibited lower mean reductions, with a mean IOP reduction of 20.6% (SD 33.5%; *n* = 29) in patients younger than 2 years and 18.0% (SD 27.0%; *n* = 27) in those aged 2–5 years. Overall, a wide variability in treatment response was observed across all age groups, as reflected by the large standard deviations (Figure 5).

No significant correlation was found between patient age and percentage IOP reduction. Spearman’s rank correlation analysis demonstrated a very weak positive association (rho = 0.059), which was not statistically significant (*p* = 0.554), indicating that age alone did not significantly influence the magnitude of IOP reduction.

Regarding glaucoma type, the greatest mean percent reduction in IOP was observed in congenital glaucoma (mean reduction: 40.0%, SD: 9.30%; absolute reduction: 21.0 mmHg, SD: 9.59), which also showed the highest success rate (100%). This was followed by juvenile glaucoma (mean reduction: 33.7%, SD: 26.8%; absolute reduction: 8.32 mmHg, SD: 5.81; success rate 80%). Moderate reductions were noted in unspecified glaucoma (mean 25.0%, SD: 21.6%; 8.0 mmHg, SD: 7.0) and inflammatory glaucoma (mean 22.3%, SD: 29.4%; 9.17 mmHg, SD: 12.1). The lowest mean percent reductions were observed in post-traumatic glaucoma (mean 21.7%, SD: 32.2%; 7.9 mmHg, SD: 11.5), multifactorial glaucoma (mean 17.9%, SD: 26.0%; 6.69 mmHg, SD: 9.29), other glaucoma types (mean 17.0%, SD: 34.1%; 7.47 mmHg, SD: 12.2), and post-cataract surgery glaucoma (mean 15.3%, SD: 47.3%; 6.5 mmHg, SD: 17.7).

Statistical analysis using the Kruskal–Wallis test revealed no significant differences between glaucoma types in terms of absolute IOP change (*p* = 0.633) and percent IOP change (*p* = 0.808).

MP-TSCPC showed a favorable safety profile, with no severe adverse events reported. The procedure was effective even in eyes with complex etiologies, including inflammatory causes or complex developmental dysgeneses. The analysis demonstrated the broad range of underlying conditions—congenital anomalies, acquired causes, and systemic syndromes—confirming the heterogeneity of pediatric secondary glaucoma. In conclusion, MP-TSCPC proved to be highly effective in lowering IOP and reducing medication burden, particularly in patients without prior surgical treatment. Its repeatability and safety profile make it a valuable therapeutic option for pediatric secondary glaucoma.

## 4. Discussion

The results of this study confirm that MP-TSCPC is an effective and safe treatment modality for pediatric patients with secondary glaucoma. A significant reduction in IOP was achieved in 69.6% of analyzed cases, with the most pronounced effect observed in patients who had not undergone prior glaucoma surgical intervention. This finding highlights the potential of MP-TSCPC as a first-line intervention in pediatric secondary glaucoma, providing substantial IOP control in surgery-naive eyes (mean reduction: 22%) and in eyes with a history of prior interventions (mean reduction: 19%).

In a prospective interventional study on 23 eyes with refractory pediatric glaucoma with at least three follow-ups up to six months [28] authors demonstrated a significant mean IOP reduction at each follow-up visit (*p* = 0.000014), with overall success rates of 56.5% at 1 month and 34.7% at 6 months. Two eyes (8.6%) required additional intervention within the first month, rising to 13 eyes (56.5%) overall. No major complications were noted. IOP reduction results reported in our study (20.8%) are comparable to those of Sivasubramanian et al. The significant *p* value suggests a similar magnitude. Importantly, the success rate reported in our study (69.6%) is higher than that reported by Sivasubramanian et al. (56.5%) at 1 month and 34.7% at 6 months. This divergence may reflect differences in study design: our analysis considered each procedure individually—including repeat treatments—versus their “per-eye” success assessment. Additionally, our longer mean follow-up (~317 days) may have permitted greater cumulative benefit, especially given our observation that repeat procedures yielded satisfactory outcomes in over 50% of cases.

Sivasubramanian et al. reported no serious adverse events, consistent with our findings. Our results add further evidence that MP-TSCPC is effective even in highly heterogeneous and complex pediatric secondary glaucoma etiologies (e.g., uveitis, aphakia, congenital anomalies). Moreover, we observed a greater response in eyes naive to previous glaucoma surgery, reinforcing earlier findings that extensive surgical history may limit MP-TSCPC efficacy.

Notably, Sivasubramanian et al. reported a decline in treatment success over time; however, direct temporal comparisons are limited by differences in study design and follow-up schedules. In our cohort, follow-up visits were not performed at standardized time points but occurred according to routine clinical practice and hospital attendance.

Nevertheless, despite these constraints, our findings support MP-TSCPC as a safe, effective, and flexible treatment option for pediatric secondary glaucoma, particularly in surgically naive eyes and in patients at high surgical risk. Collected data indicate that MP-TSCPC enables a reduction in IOP that remains stable over time, due to the possibility of repeating the procedure when clinically required.

The study of Garcia et al., in which eyes with prior glaucoma surgery experienced a greater reduction in IOP compared to those without prior surgical interventions [29], diverges from our findings, where eyes without prior surgeries showed a greater mean IOP reduction compared to eyes with a history of surgical treatment. A key difference between the two studies is the age of the participants. In our study, the patient cohort consisted exclusively of a pediatric population, with ages ranging from 4 months to 17 years, whereas in Garcia et al.’s study, the age of patients ranged from 13 to 94 years. These demographic differences may have contributed to the variations in treatment response observed between the two studies.

Tekeli et al. evaluated the efficacy of MP-TSCPC in a predominantly adult population with primary open-angle glaucoma (POAG), pseudoexfoliative glaucoma (PEXG), and other secondary glaucomas [30]. In their cohort of 74 eyes, the mean preoperative IOP was 28.1 ± 8.6 mmHg, which decreased to 18.9 ± 5.6 mmHg at the final follow-up, corresponding to a 32.7% reduction. Treatment success, defined as ≥20% IOP reduction without additional glaucoma surgery, was achieved in 70.3% of eyes at 6 months and 64.9% at 12 months. In contrast, our pediatric population comprising 59 children (68 eyes) with a heterogeneous spectrum of secondary glaucomas experienced a mean IOP reduction from 34.0 mmHg to 25.8 mmHg, a 20.8% decrease. While this percentage reduction is somewhat lower than that reported by Tekeli and Köse, our study demonstrated a comparable success rate of 69.6%, despite the greater variety of secondary glaucoma causes and surgically pretreated pediatric eyes. Furthermore, while Tekeli et al. observed complications such as hypotony and prolonged inflammation, no severe adverse events were reported in our study group.

The reduction in IOP, coupled with reduction in the number of anti-glaucoma medications in 33.3% of cases, with an overall decrease in the mean number of medications from 2.42 to 2.02 per patient (*p* < 0.05), suggests that MP-TSCPC not only lowers IOP but also has the potential to decrease the pharmacological burden in pediatric patients with secondary glaucoma. Ariga et al. (2021), in their study which evaluated MP-TSCP in refractory glaucoma, in comparison to ours, revealed notable differences in the extent of medication reduction. Ariga et al. reported a decrease in the mean number of medications from 3.6 to 2.7, with 57% of patients requiring fewer medications postoperatively [31]. While both studies demonstrated a statistically significant reduction in medication usage, the extent of reduction in our cohort was more modest. This discrepancy may be attributed to differences in study populations, as Ariga et al. included predominantly adult patients with refractory glaucoma, whereas our study focused on pediatric patients with secondary glaucoma. The pediatric population presents unique challenges, including variability in disease etiology, altered pharmacokinetics, and a higher likelihood of progressive pathology, which may impact the extent to which medications can be safely reduced [32].

According to Sharifipour et al., children diagnosed with glaucoma at a younger age, those undergoing their first glaucoma surgery earlier, and those with systemic comorbidities are at an increased risk of disease progression [33]. This aligns with observations from Kirwan et al., who found that the success rate in their pediatric sample was lower than that typically reported in adult patients. They suggested that this could be due to the faster recovery of ciliary body function in younger eyes, a result of tissue regeneration, which may lead to a quicker return of IOP and contribute to less sustained treatment outcomes in pediatric patients [34].

The possibility of repeating the procedure in cases where the initial treatment did not achieve satisfactory IOP control is one of MP-TSCPC’s benefits. In our study a group of repeated MP-TSCPC procedures consisted of 28 eyes, with a success rate of 57.1%. This highlights the potential of MP-TSCPC as a flexible and adjustable treatment strategy for patients with refractory glaucoma, allowing for individualized management based on the patient’s response to therapy.

It is important to note that pediatric secondary glaucoma represents a highly heterogeneous group of conditions, and our cohort included a wide spectrum of underlying etiologies such as uveitis, aphakia, congenital anomalies, and post-surgical cases. Due to this heterogeneity and the limited number of cases in each etiologic subgroup, we were unable to perform detailed stratified analyses of MP-TSCPC efficacy by specific glaucoma subtype. Therefore, while overall treatment outcomes were favorable, the response to MP-TSCPC may vary across different subgroups, and these differences could not be fully evaluated in our study.

It could also be used as a bridging procedure to other, more effective hypotensive surgeries. When comparing our results with those reported by Hooshmand et al., notable differences in success rates can be observed. In their study, the success rate increased from 23.5% after the initial procedure to 44.1% following the repeated procedure (*p* = 0.123) [35]. Our study demonstrated a higher overall success rate of 57.1% for repeated MP-TSCPC in pediatric patients with secondary glaucoma. This suggests that, in our cohort, repeated MP-TSCPC may provide a greater benefit in terms of IOP control compared to the outcomes observed in adult patients. However, differences in patient demographics, underlying pathology, and study design must be considered when interpreting these results.

Findings from other studies support the role of MP-TSCPC as a minimally invasive yet effective approach to glaucoma management, offering a high level of safety. MP-TSCPC has also been recognized as a safer alternative to the CW-TSCPC procedure, with a lower incidence of sight-threatening complications [36]. MP-TSCPC is still associated with certain adverse effects, including persistent inflammation lasting more than three months, a decline in best-corrected visual acuity, early, late, and persistent hypotony, as well as cystoid macular edema. Complications that occur less frequently include corneal edema, IOP spikes, severe inflammation with fibrin in the pupil, transient hyphema, persistent mydriasis, scleral thinning, and serous choroidal detachment [37,38,39]. In our study, none of the patients experienced any adverse effects. This absence of complications highlights the potential advantages of MP-TSCPC in minimizing post-procedural risks while maintaining therapeutic efficacy, preserving vision and quality of life. Our results contribute to the growing body of evidence supporting MP-TSCPC as a well-tolerated and effective treatment option [40].

## 5. Limitations

This study has several limitations that should be acknowledged. First, its retrospective design inherently limits the ability to control for confounding variables and introduces the risk of selection and information bias. Data collection depended on the accuracy and completeness of medical records, which may have affected the consistency of some variables.

Second, this was a single-center study, which may limit the generalizability of the findings to broader pediatric populations. Treatment protocols, surgical expertise, and follow-up strategies may differ across institutions.

Third, the study population was characterized by marked heterogeneity in terms of glaucoma etiology. The analyzed cohort included congenital anomalies, inflammatory conditions, post-surgical cases, traumatic causes, and systemic syndromes. Although this reflects real-world clinical practice, such heterogeneity may have influenced treatment response and limited the ability to draw etiology-specific conclusions. Moreover, due to the small number of cases within each specific glaucoma subtype, we could not analyze the efficacy of MP-TSCPC in a stratified manner. This limitation prevents us from determining whether the observed treatment success was consistent across all etiologic subgroups or predominantly driven by particular types of secondary glaucoma

Additionally, gender-based analysis was not performed. Potential sex-related differences in treatment response or disease progression were therefore not assessed and should be explored in future studies.

Another limitation is the relatively variable and in some cases short follow-up period, which may not fully capture long-term treatment efficacy, late complications, or the durability of repeated MP-TSCPC procedures.

Finally, although repeatability of the procedure was analyzed, the sample size for repeated treatments was limited, reducing the statistical power of subgroup analyses.

Future prospective, multicenter studies with longer follow-up periods and more homogeneous subgroups are needed to better define predictors of treatment success and optimize patient selection.

## 6. Future Directions

The promising results of MP-TSCPC in pediatric secondary glaucoma suggest several potential future directions. This minimally invasive and repeatable procedure may increasingly serve as an adjunct or alternative to conventional pharmacotherapy, potentially reducing the long-term reliance on topical medications and their associated side effects. In combination with other therapeutic modalities, including selective laser treatments and modern surgical interventions, MP-TSCPC may offer a more tailored, stepwise management approach, especially for patients with complex or refractory glaucoma. Future prospective, multicenter studies with longer follow-up are warranted to evaluate the long-term efficacy, safety, and optimal integration of MP-TSCPC into comprehensive treatment strategies. Furthermore, investigations into patient-specific predictors of response could enable more precise, individualized treatment planning, ultimately improving visual outcomes and quality of life for pediatric patients.

## 7. Conclusions

MP-TSCPC is an effective and well-tolerated treatment option for pediatric patients with secondary glaucoma. This study highlights its potential not only in achieving meaningful IOP reduction but also in decreasing the need for anti-glaucoma medications, thereby lowering the overall treatment burden. Notably, the greatest therapeutic benefit was observed in treatment-naive eyes, suggesting that MP-TSCPC may be particularly useful as an early intervention. One of the key advantages of MP-TSCPC is its adaptability, allowing for repeated procedures when initial treatment does not provide sufficient IOP control. This flexibility makes it a valuable option for managing refractory cases while maintaining a favorable safety profile. MP-TSCPC offers a minimally invasive approach with a reduced risk of severe complications, making it especially relevant in the pediatric population.

Cover letter: Secondary glaucoma in children results from congenital or acquired ocular abnormalities, systemic diseases, or syndromes. These conditions impair aqueous humor outflow despite an open iridocorneal angle, causing elevated IOP. MP-TSCPC reduces aqueous humor production by targeting the ciliary body and restores the aqueous humor’s circulation balance. The aim of our study was to evaluate the safety and efficacy of MP-TSCPC in pediatric secondary glaucoma. Our manuscript includes a study in pediatric patients.

## Data Availability

The datasets used and/or analyzed during the current study are available from the corresponding author on reasonable request.

## Abbreviations

The following abbreviations are used in this manuscript:

MP-TSCPC	Micropulse Transsceral Cyclophotocoagulation
CW-TSCPC	Continuous -Wave Transsceral Cyclophotocoagulation
IOP	Intraocular pressure

## References

[1] Ezinne N.E., Shittu O., Ekemiri K.K., Kwarteng M.A., Tagoh S., Ogbonna G., Mashige K.P. (2022). Visual Impairment and Blindness among Patients at Nigeria Army Eye Centre, Bonny Cantonment Lagos, Nigeria. Healthcare.

[2] Sen S., Zeppieri M., Tripathy K. (2024). Uveitis Glaucoma Hyphema Syndrome. StatPearls.

[3] Razeghinejad R., Lin M.M., Lee D., Katz L.J., Myers J.S. (2020). Pathophysiology and management of glaucoma and ocular hypertension related to trauma. Surv. Ophthalmol..

[4] Buffault J., Brignole-Baudouin F., Reboussin É., Kessal K., Labbé A., Mélik Parsadaniantz S., Baudouin C. (2022). The Dual Effect of Rho-Kinase Inhibition on Trabecular Meshwork Cells Cytoskeleton and Extracellular Matrix in an In Vitro Model of Glaucoma. J. Clin. Med..

[5] Brandt J.D., Shankar S.P. (2023). Glaucoma Following Cataract Surgery in Children—Finally, a Clue. JAMA Ophthalmol..

[6] Shen W.C., Huang B.Q., Yang J. (2023). Regulatory mechanisms of retinal ganglion cell death in normal tension glaucoma and potential therapies. Neural Regen. Res..

[7] Khandwala N.S., Ramappa M., Edward D.P., Mocan M.C. (2023). Axenfeld-Rieger syndrome in the pediatric population: A review. Taiwan J. Ophthalmol..

[8] Dingenen E., Segers D., De Maeseneer H., Van Gysel D. (2024). Sturge–Weber syndrome: An update for the pediatrician. World J. Pediatr..

[9] Akabane N., Hamanaka T. (2003). Histopathological study of a case with glaucoma due to Sturge–Weber syndrome. Jpn. J. Ophthalmol..

[10] Reis L.M., Semina E.V. (2011). Genetics of anterior segment dysgenesis disorders. Curr. Opin. Ophthalmol..

[11] Carrabba N., Zhaver D., Blieden L.S. (2022). Surgical Management of Secondary Pediatric Glaucoma. Int. Ophthalmol. Clin..

[12] Wallace D.K., Plager D.A., Snyder S.K., Raiesdana A., Helveston E.M., Ellis F.D. (1998). Surgical results of secondary glaucomas in childhood. Ophthalmology.

[13] Younus M., Schachar R.A., Zhang M.I.N., Sultan M.B., Tressler C.S., Huang K., Xu W., Klein M., Platt R.W., Mukherjee N. (2018). A Long-term Safety Study of Latanoprost in Pediatric Patients With Glaucoma and Ocular Hypertension: A Prospective Cohort Study. Am. J. Ophthalmol..

[14] Urban B., Bakunowicz-Łazarczyk A., Mrugacz M., Oziębło-Kupczyk M. (2004). Skuteczność latanoprostu w jaskrze wieku dzieciecego [The effectiveness of latanoprost for the treatment of pediatric glaucoma]. Klin. Oczna.

[15] Heinz C., Pleyer U., Ruokonnen P., Heiligenhaus A. (2008). Sekundärglaukom bei Kindern mit Uveitis [Secondary glaucoma in childhood uveitis]. Ophthalmologe.

[16] Skov A.G., Rives A.S., Freiberg J., Virgili G., Azuara-Blanco A., Kolko M. (2022). Comparative efficacy and safety of preserved versus preservative-free beta-blockers in patients with glaucoma or ocular hypertension: A systematic review. Acta Ophthalmol..

[17] Schlote T., Derse M., Zierhut M. (2000). Transscleral diode laser cyclophotocoagulation for refractory glaucoma secondary to inflammatory eye diseases. Br. J. Ophthalmol..

[18] Muralidharan S., Kumar S., Ichhpujani P., Dhillon H.K. (2022). Quality of life in glaucoma patients: Comparison of medical therapy, trabeculectomy, and glaucoma drainage device surgery. Indian J. Ophthalmol..

[19] Walters T.R., Kothe A.C., Boyer J.L., Usner D.W., Lopez K., Duquesroix B., Fechtner R.D., Navratil T. (2022). A Randomized, Controlled Comparison of NCX 470 (0.021%, 0.042%, and 0.065%) and Latanoprost 0.005% in Patients With Open-angle Glaucoma or Ocular Hypertension: The Dolomites Study. J. Glaucoma.

[20] Song J., Song A., Palmares T., Song M. (2013). Selective laser trabeculoplasty success in pediatric patients with glaucoma: Two case reports. J. Med. Case Rep..

[21] Zhou R., Sun Y., Chen H., Sha S., He M., Wang W. (2021). Laser Trabeculoplasty for Open-Angle Glaucoma: A Systematic Review and Network Meta-Analysis. Am. J. Ophthalmol..

[22] Greslechner R., Helbig H., Spiegel D. (2022). Sekundäre Offenwinkelglaukome: Uveitische Sekundärglaukome, Steroidglaukom, posttraumatische/postoperative Glaukome, tumorbedingte Glaukome und Glaukome im Rahmen eines erhöhten episkleralen Venendrucks [Secondary open-angle glaucoma: Uveitic secondary glaucoma, steroid-induced glaucoma, posttraumatic and postoperative glaucoma, tumor-related glaucoma and glaucoma due to elevated episcleral venous pressure]. Ophthalmologe.

[23] Seixas R.C.S., Russ H.H.A., Maestrini H.A., Balbino M., Fernandes T.A.P., Lima N.V.D.A., Lopes N.L.V., Neto T.D.S.R. (2024). Slow coagulation versus micropulse transscleral cyclophotocoagulation for refractory childhood glaucoma. Eur. J. Ophthalmol..

[24] Desmettre T.J., Mordon S.R., Buzawa D.M., Mainster M.A. (2006). Micropulse and continuous wave diode retinal photocoagulation: Visible and subvisible lesion parameters. Br. J. Ophthalmol..

[25] Barac R., Vuzitas M., Balta F. (2018). Choroidal thickness increase after micropulse transscleral cyclophotocoagulation. Rom. J. Ophthalmol..

[26] Maestri F., Legrand M., Da Cunha E., Best A.L., Benichou J., Barreau E., Labetoulle M., Rousseau A. (2021). Cycloaffaiblisement au laser diode micropulsé: Une technique efficace, mais dont la stratégie reste à définir [Micropulsed diode laser transscleral cyclophotocoagulation: An effective technique whose role remains to be defined]. J. Fr. Ophtalmol..

[27] Nirappel A., Klug E., Neeson C., Chachanidze M., El Helwe H., Hall N., Chang T.C., Shen L.Q., Solá-Del Valle D. (2023). Transscleral vs endoscopic cyclophotocoagulation: Safety and efficacy when combined with phacoemulsification. BMC Ophthalmol..

[28] Sivasubramanian R., Siddharth K., Senthilkumar V.A., Mani I., Rajendrababu S. (2025). Safety and efficacy of micropulse in pediatric eyes with refractory glaucoma. Indian J. Ophthalmol..

[29] Garcia G.A., Nguyen C.V., Yelenskiy A., Akiyama G., McKnight B., Chopra V., Lu K., Huang A., Tan J.C., Francis B.A. (2019). Micropulse Transscleral Diode Laser Cyclophotocoagulation in Refractory Glaucoma: Short-Term Efficacy, Safety, and Impact of Surgical History on Outcomes. Ophthalmol. Glaucoma.

[30] Tekeli O., Köse H.C. (2021). Outcomes of micropulse TSCPC in POAG, PXG, and secondary glaucoma. Eur. J. Ophthalmol..

[31] Ariga M., Nivean, Nivean P.D., Madanagopalan V.G., Mohan S. (2021). Micropulse trans-scleral diode laser cyclophotocoagulation in refractory glaucoma: An initial experience in Indian eyes. Int. Ophthalmol..

[32] Lu H., Rosenbaum S. (2014). Developmental pharmacokinetics in pediatric populations. J. Pediatr. Pharmacol. Ther..

[33] Sharifipour F., Arasteh E., Hajizadeh M., Mahdian-Rad A., Mirdehghan M.S. (2021). Progression in pediatric glaucoma: Lessons learnt from 8 years’ follow-up. Med. Hypothesis Discov. Innov. Ophthalmol..

[34] Kirwan J.F., Shah P., Khaw P.T. (2002). Diode laser cyclophotocoagulation: Role in the management of refractory pediatric glaucomas. Ophthalmology.

[35] Hooshmand S., Voss J., Hirabayashi M., McDaniel L., An J. (2022). Outcomes of initial and repeat micro-pulse transscleral cyclophotocoagulation in adult glaucoma patients. Ther. Adv. Ophthalmol..

[36] Zemba M., Dumitrescu O.M., Vaida F., Dimirache E.A., Pistolea I., Stamate A.C., Burcea M., Branisteanu D.C., Balta F., Barac I.R. (2022). Micropulse vs. continuous wave transscleral cyclophotocoagulation in neovascular glaucoma. Exp. Ther. Med..

[37] Abdelmassih Y., Tomey K., Khoueir Z. (2021). Micropulse Transscleral Cyclophotocoagulation. J. Curr. Glaucoma. Pract..

[38] Dhanireddy S., Dhanireddy S., Yin H.Y., Dosakayala N., Kurochkin P., Gupta N., Cheng A.M., Fechtner R., Alpert S. (2020). Severe Inflammation and Hyphema After Micropulse Diode Transscleral Cyclophotocoagulation. J. Glaucoma.

[39] Williams A.L., Moster M.R., Rahmatnejad K., Resende A.F., Horan T., Reynolds M., Yung E., Abramowitz B., Kuchar S., Waisbourd M. (2018). Clinical Efficacy and Safety Profile of Micropulse Transscleral Cyclophotocoagulation in Refractory Glaucoma. J. Glaucoma.

[40] Abdelrahman A.M., El Sayed Y.M. (2018). Micropulse vs continuous wave TSCPC in refractory pediatric glaucoma. J. Glaucoma.

